# From Petri Dish to Primitive Heart: How IVF Alters Early Cardiac Gene Networks and Epigenetic Landscapes

**DOI:** 10.3390/biomedicines13082044

**Published:** 2025-08-21

**Authors:** Charalampos Voros, Georgios Papadimas, Marianna Theodora, Despoina Mavrogianni, Diamantis Athanasiou, Ioakeim Sapantzoglou, Kyriakos Bananis, Antonia Athanasiou, Aikaterini Athanasiou, Charalampos Tsimpoukelis, Ioannis Papapanagiotou, Dimitrios Vaitsis, Aristotelis-Marios Koulakmanidis, Maria Anastasia Daskalaki, Vasileios Topalis, Nikolaos Thomakos, Panagiotis Antsaklis, Fotios Chatzinikolaou, Dimitrios Loutradis, Georgios Daskalakis

**Affiliations:** 1 Department of Obstetrics and Gynecology, ‘Alexandra’ General Hospital, National and Kapodistrian University of Athens, 80 Vasilissis Sofias Avenue, 11528 Athens, Greece; martheodr@gmail.com (M.T.); depy.mavrogianni@yahoo.com (D.M.); kimsap1990@hotmail.com (I.S.); tsimpoukelischa@gmail.com (C.T.); aristoteliskoulak@gmail.com (A.-M.K.); md181341@students.euc.ac.cy (M.A.D.); thomakir@hotmail.com (N.T.); panosant@gmail.com (P.A.); gdaskalakis@yahoo.com (G.D.); 2 Athens Medical School, National and Kapodistrian University of Athens, 15772 Athens, Greece; dr.georgepapadimas@gmail.com (G.P.); gpapamd@hotmail.com (I.P.); vaitsisdim@gmail.com (D.V.); vtopalismd@gmail.com (V.T.); loutradi@otenet.gr (D.L.); 3 IVF Athens Reproduction Center, 15123 Maroussi, Greece; diamathan16@gmail.com (D.A.); antoathan16@gmail.com (A.A.); diamathan17@gmail.com (A.A.); 4 King’s College Hospitals NHS Foundation Trust, London SE5 9RS, UK; kyriakos.bananis@nhs.net; 5 Laboratory of Forensic Medicine and Toxicology, School of Medicine, Aristotle University of Thessaloniki, 54124 Thessaloniki, Greece; fotischatzin@auth.gr; 6 Fertility Institute-Assisted Reproduction Unit, Paster 15, 11528 Athens, Greece

**Keywords:** in vitro fertilization, cardiogenesis, congenital heart defects, epigenetic regulation, cardiac transcription factors

## Abstract

Numerous infants have been conceived by in vitro fertilization (IVF) and other assisted reproductive technologies (ART). Increasing evidence indicates that these approaches induce minor alterations in molecules during the initial phases of embryogenesis. This narrative review examines the molecular pathophysiology of embryonic cardiogenesis in the context of assisted reproductive technology, emphasizing transcriptional and epigenetic regulation. Essential transcription factors for cardiac development, including NKX2-5, GATA4, TBX5, ISL1, MEF2C, and HAND1/2, play a crucial role in mesodermal specification, heart tube formation, and chamber morphogenesis. Animal models and human preimplantation embryos have demonstrated that ART-related procedures, including gamete micromanipulation, supraphysiological hormone exposure, and extended in vitro culture, can alter the expression or epigenetic programming of these genes. Subsequent to ART, researchers have identified anomalous patterns of DNA methylation, alterations in histones, and modifications in chromatin accessibility in cardiogenic loci. These alterations indicate that errors occurred during the initial reprogramming process, potentially resulting in structural congenital heart abnormalities (CHDs) or modifications in cardiac function later in life. Analysis of the placental epigenome in babies conceived using assisted reproductive technology reveals that imprinted and developmental genes critical for cardiac development remain dysfunctional. This review proposes a mechanistic theory about the potential subtle alterations in the cardiogenic gene network induced by ART, synthesizing findings from molecular embryology, transcriptomics, and epigenomics. Understanding these molecular issues is crucial not only for enhancing ART protocols but also for evaluating the cardiovascular risk of children conceived by ART postnatally and for early intervention.

## 1. Introduction

### 1.1. Background and Clinical Rationale

In vitro fertilization (IVF) and intracytoplasmic sperm injection (ICSI), two forms of assisted reproductive technologies (ART), have transformed infertility treatment and have contributed to the birth of over 9 million children globally [1]. Concerns are increasing regarding the potential unintended biological consequences of ART on early embryonic development, fetal organogenesis, and the long-term health of children, despite its evident success [2]. A critical issue is the association between ART and congenital malformations, particularly congenital heart abnormalities (CHDs), which represent the most prevalent form of birth anomaly globally [3].

Numerous population-based studies and meta-analyses have consistently demonstrated that children produced via ART exhibit a higher incidence of congenital heart abnormalities (CHDs) compared to those conceived naturally [4]. Boulet et al. (2016) conducted a comprehensive analysis across three U.S. states, revealing that infants produced via ART had a 28% increased adjusted risk of non-chromosomal birth anomalies. Cardiovascular abnormalities constituted a significant aspect of this cohort [5]. Although the absolute risk remains minimal, the recurrent occurrence in numerous experiments suggests an underlying biological susceptibility that warrants further investigation.

The mechanisms by which ART induces these developmental issues remain unclear. The preimplantation phase is very significant for epigenetic reprogramming and lineage specification. This is when assisted reproductive technology treatments such as ovarian hyperstimulation, oocyte retrieval, in vitro fertilization, embryo culture, micromanipulation (including ICSI), and embryo transfer occur [6,7]. In this period, the DNA within the mammalian embryo undergoes widespread demethylation, followed by a subsequent remethylation that establishes unique gene expression patterns for each cell type [8]. ART removes the embryo from the meticulously regulated environment of the maternal reproductive system. It then exposes the embryo to conditions that exceed physiological parameters, temperature fluctuations, and mechanical stress. Each of these factors can alter the embryo’s epigenetic landscape.

Initial alterations in epigenetics have been associated with various developmental issues, including imprinting disorders (such as Beckwith–Wiedemann syndrome and Silver–Russell syndrome), reduced birth weight, modifications in placental function, and an increased susceptibility to cardiovascular disease and metabolic disorders in adolescence and adulthood [9]. The heart is among the first organs to develop and function throughout embryogenesis. It originates from mesodermal progenitors that exhibit high responsiveness to transcriptional, epigenetic, and morphogenetic cues [10]. Any alteration in these rigorously regulated signaling networks during the initial days of development could affect the commitment to cardiac lineage, morphogenesis, or functional maturation [11].

The placenta and heart develop similarly and are regulated by the same molecules, including genes associated with angiogenesis, extracellular matrix remodeling, and oxygen sensing [12]. Abnormal placental epigenetics, prevalent in ART pregnancies, may influence cardiac development by altering nutrient delivery, hormone production, and vascular signaling. This establishes a two-hit paradigm of intrauterine cardiovascular programming [13].

Concurrently, ART frequently occurs alongside parental subfertility, advanced maternal or paternal age, and preexisting medical or metabolic conditions. All of these factors may influence embryonic development individually or in conjunction with one another [14]. Distinguishing between the effects of ART procedures and the influences of infertility is challenging yet crucial for determining causality. These maternal and preconceptional factors can independently induce epigenetic alterations in gametes and early embryos, irrespective of assisted reproductive technology. Therefore, it is essential to distinguish the direct impacts of ART techniques from those of fundamental infertility, parental age, or concomitant conditions for accurate interpretation.

Integrating epidemiological observations, experimental models, and molecular biology indicates a compelling rationale: ART may influence the embryo’s cardiovascular development by altering gene expression and rearrangement during the initial stages of the process [15]. Due to the heart’s complexity, unique morphology, and vital role in sustaining life, it is essential to comprehend these systems to safeguard children’s health and improve ART protocols and embryo management prior to implantation [16].

The primary objective of this review is to elucidate the molecular factors contributing to the increased incidence of cardiac anomalies in embryos created via ART. We examine the impact of ART on the expression, regulation, and epigenetic integrity of crucial cardiac transcription factors and signaling pathways that govern early cardiac development.

### 1.2. Cardiogenesis: Genetic Networks and Molecular Pathways

The development of the mammalian heart is among the most intricate and exact processes occurring throughout embryonic growth. The heart is the initial organ to function in the embryo. The formation commences promptly following gastrulation, as mesodermal cells are designated to differentiate into cardiac cells and migrate to establish the cardiac crescent [17]. This process is morphogenetically intricate and meticulously regulated at the molecular level through a hierarchical interplay of signaling pathways, transcription factors, and chromatin dynamics [18]. Dynamic regulatory networks must accurately activate lineage-specific genes and deactivate non-cardiac programs for healthy heart development. Any alteration to this molecular blueprint, particularly during the initial phases of development, can increase the embryo’s susceptibility to structural cardiac anomalies or enduring functional issues [19].

Two categories of mesodermal cells generate cardiac progenitors: the first heart field (FHF), predominantly forming the left ventricle and atria, and the second or anterior heart field (AHF), which constitutes the right ventricle and outflow tract [20]. Essential extracellular signaling molecules, including bone morphogenetic proteins (BMPs), WNTs, fibroblast growth factors (FGFs), and members of the TGF-β superfamily, delineate these progenitor pools [21,22]. These morphogens initiate transcriptional processes that establish the identity of the heart and regulate cellular positioning, growth, and transformation [23]. ISL1 is an additional early cardiac transcription factor, and Section 2.1 elaborates extensively on its molecular functions [24,25].

As cardiac progenitors commit to the myocardial lineage, they activate a fundamental network of transcription factors. NKX2-5, GATA4, and TBX5 are among the primary and most crucial transcription factors in cardiac development. These variables together regulate myocardial commitment, chamber specification, and the activation of structural cardiac genes. Section 2.1 delineates their precise molecular activities, their interactions, and the manner in which ART enhances their susceptibility [26,27,28,29].

TBX5 is a transcription factor belonging to the T-box family. It subsequently regulates gene expression in specific chambers, particularly in the left ventricle and atria. It collaborates with NKX2-5 and GATA4 to activate structural genes such as MYH6, ACTC1, and TNNT2 [30]. Pathogenic alterations in TBX5 are the primary genetic etiology of Holt–Oram syndrome, a disorder characterized by limb malformations and cardiac septal defects [31]. As elucidated in Section 2.1, MEF2C integrates TGFβ–Smad signaling during the formation of the right ventricle and outflow tract [32,33]. Section 2.1 provides a summary of the functions of HAND1 and HAND2 in defining chambers and laterality [32,34,35].

Cardiogenesis is significantly influenced by chromatin-level regulation alongside transcription factors. Epigenetic mechanisms, such as histone modifications, nucleosome positioning, and DNA methylation, regulate the accessibility of cis-regulatory regions, including enhancers and promoters [36]. Histone modifications such as H3K27ac and H3K4me1 signify active cardiac enhancers in the developing heart [19]. GATA4, MEF2C, and TBX5 frequently utilize these enhancers. These enhancers constitute cardiac-specific super-enhancer clusters that facilitate the transcription of genes regulating contractility, calcium management, and cytoskeletal architecture at elevated levels. Investigators have demonstrated that epigenetic regulators such as HDAC3, DNMT3A, and KDM6A are essential for proper cardiac development [37,38]. Malfunctioning regulators may result in significant congenital anomalies.

Cardiogenesis is regulated by many signaling mechanisms that converge to alter gene expression and the activity of progenitor cells. For the early patterning of mesoderm to occur, BMP signaling must take place [39]. It also activates GATA4 and NKX2-5. FGF signaling, particularly via FGF8 and FGF10, regulates the growth and differentiation of the SHF. The WNT signaling pathway has two phases: initially facilitating mesoderm formation, followed by a necessary cessation to enable heart development [10]. The Notch and TGFβ/Smad pathways facilitate cellular decision-making in the heart and endocardium. They assist in trabeculation, valve formation, and the organization of the conduction system [40].

The forkhead transcription factor FOXH1 is an extensively researched entity that functions as a nuclear regulator of TGFβ-Smad signaling. FOXH1 collaborates with NKX2-5 to directly bind and activate MEF2C expression in the anterior cardiac area [41]. Alterations in FOXH1 or its absence disrupt the creation of the outflow tract and the development of the right ventricle, akin to the effects observed in the absence of MEF2C. This axis illustrates the direct impact of extracellular signals on transcriptional regulation. This may render cells more susceptible to extrinsic stimuli, such as the manipulation of embryos in vitro [42].

The process of cardiogenesis is regulated by a hierarchical arrangement of transcriptional circuits and epigenetic alterations that function in conjunction with localized signaling inputs. These systems are highly vulnerable to alterations, particularly when lineage commitment is nascent. When embryos are cultivated externally to the maternal reproductive system and subjected to mechanical, nutritional, and oxidative stress, as occurs in ART, these gene regulatory networks may undergo minor yet significant alterations. The early cardiac field may be especially susceptible to ART-induced developmental reprogramming due to its dependence on precise transcriptional and epigenetic regulation.

### 1.3. Epigenetic Dysregulation in Embryos Induced by Assisted Reproductive Technology

The preimplantation phase is a period during which the genomes of both parents undergo a comprehensive reset. This encompasses the substitution of protamine for histone, both active and passive DNA demethylation, and the dynamic reconfiguration of histone modifications such as H3K4me1, H3K27ac, H3K9me3, and H3K27me3 [43]. During this period, the zygote initiates ZGA by orchestrating the activation of transcriptional programs crucial for lineage specification. During this critical period, ART procedures such as IVF, ICSI, IVM, and extended IVC subject the embryo to non-physiological oxygen tension, synthetic media devoid of maternal oviductal components, pH fluctuations, and mechanical stresses. These alterations can disrupt the global 5mC/5hmC equilibrium and reduce enhancer priming at distant regulatory elements. They can also interfere with nucleosome positioning at key cardiac transcription factor sites. In IVF mouse embryos, this is accompanied by delayed activation of lgf1, Pafahba1, and αTgf, insufficient expression of TE markers such as Eomes and Soc3, and downregulation of Hbegf [44].

Profiling the methylome and transcriptome of ART-derived blastocysts and placentas substantiates these findings. Employing RRBS, EPIC arrays, and WGBS to delineate global methylation in ART embryos and tissues reveals that DMRs are predominantly found in ICRs and cardiac-specific enhancers [45,46]. Genes such as H19, Igf2, Peg3, Mest, and Dlk1 exhibit either insufficient or excessive methylation, or they undergo allele relaxation, disrupting the patterns of monoallelic expression. The imprinting errors are associated with alterations in the expression of Trim28, Zfp57, and Zac1, which are crucial for ICR silencing. This is particularly evident in ART placentas, which have reduced levels of Notch3, Dlk1, and Cdkn1c, all of which are crucial for angiogenesis and the functionality of the cardiac–placental axis [47,48]. Investigations into IVF/ICSI embryos indicate a constant decline in the quantity of TE cells, accompanied by a diminished transcriptional disparity between TE and ICM, implying that lineage commitment is malfunctioning. The findings indicate that early ART-induced alterations adversely affect both embryonic and extraembryonic compartments critical for cardiac development.

Single-cell RNA-seq, ATAC-seq, and ChIP-seq analyses of ART embryos and cardiac progenitors derived from stem cells have revealed that numerous mesodermal and heart-specific transcription factors are malfunctioning [49]. In ART embryos, the expression levels and enhancer occupancy of genes such as Tbx5, Mef2c, Hand1, Isl1, and Nkx2-5 are diminished and dysfunctional. In mouse ART embryos, enhancers associated with Gata4, Mef2c, and Tbx20 in cardiac mesoderm exhibit alterations in H3K27ac intensity and chromatin accessibility [50]. Comparable analyses have not been performed in human ART embryos, and this statement reflects translational extrapolation. Concurrently, regulatory ncRNAs, including miR-1, miR-133a, miR-208a, and miR-499, exhibit dysfunction in both cardiac progenitors and fetal hearts derived from ART models. Typically, these miRNAs target anti-cardiogenic transcription factors such as Sox6, Hdac4, and Snai1, which assist in regulating the expression of sarcomeric genes and the development of cardiomyocytes [51]. If they malfunction, they may impede CM’s ability to differentiate, hinder the contraction of the heart muscle, and disrupt the organization of the cytoskeleton.

The stress induced by ART also impacts the epigenetic regulators directly. Embryos treated with IVC exhibit aberrant expression of Dnmt3a, Dnmt3b, Tet1, Hdac3, Kdm6a, and Ezh2. These enzymes are responsible for the synthesis or degradation of 5mC/5hmC patterns and the regulation of bivalent chromatin states at cardiac genes [52]. For instance, the loss of Dnmt3a function results in the derepression of non-cardiac lineage genes. Depletion of Hdac3 renders compact chromatin at cardiac enhancers ineffective [53]. In ART models, aberrant expression of Kdm6a, an H3K27me3 demethylase, has been associated with alterations in the activation of Mesp1, Gata4, and Foxh1 targets. This indicates that the processes responsible for committing mesodermal cells are disrupted. Enhancer bivalency, characterized by H3K4me1/H3K27me3 at poised cardiac loci, frequently exhibits instability in ART embryos. This may lead to the premature activation or complete inactivation of cardiogenic gene regulatory networks [54].

Furthermore, ART-induced alterations influence the three-dimensional chromatin architecture that regulates cardiac gene expression. Hi-C and Capture-C studies in murine ART embryos indicate that the TAD boundaries around Nkx2-5, Isl1, and Mef2c may have been altered, thereby diminishing the efficacy of enhancer–promoter looping [55,56]. No equivalent high-resolution chromatin conformation mapping has yet been reported in human ART embryos. During SHF deployment and OFT formation, improperly folded TADs and CTCF binding distant from super-enhancers may impede transcriptional activity. The alterations in chromatin structure may explain why ART-derived mouse embryos resemble mutants of critical cardiac transcription factors or epigenetic enzymes [57].

ART influences various aspects of transcriptional regulation during the initial phases of embryonic development. These include alterations in DNA methylation, histone post-translational modifications, non-coding RNA circuits, the three-dimensional architecture of the genome, and transcription factor co-occupancy at cardiac cis-regulatory elements [58]. ART may disrupt SHF/FHF specification and CM lineage integrity by interfering with ZGA, mesodermal induction, enhancer accessibility, and transcription factor hierarchies. This molecular instability is particularly concerning for high-order regulators such as Nkx2-5, Gata4, Mef2c, and Hand2, whose expression levels and temporal regulation are critical for cardiac development [59]. During this period, epigenetic memory may be established, persisting throughout later stages of development. This may result in CHDs or subclinical cardiac anomalies observed in infants conceived with ART. This proof elucidates how ART approaches can alter cardiac development via epigenetic and transcriptional reconfiguration.

It is essential to acknowledge that certain epigenetic modifications linked to ART may be influenced or intensified by contemporaneous parental factors, such as advanced maternal age, hormonal disorders connected to subfertility, or metabolic diseases. These factors can alter the epigenetic modifications in gametes prior to ART, increasing their susceptibility to ART-specific stresses. This interaction may clarify the variability in ART outcomes and suggests that categorizing research by these confounding factors is crucial for accurate mechanistic interpretation.

While many of the mechanistic insights presented in this review are derived from a rich body of preclinical research, it is important to distinguish between evidence confirmed in human ART contextsand findings extrapolated from animal models or in conceived placentas, differential expression od DNA methyltranferases in human preimplantation embryos, and associations between culture media composition and specific histone modification patterns-are supported by direct analysis of clinical samples and provide robust translation relevance. Conversely, several observations, including the disruption of TAD boundaries, enhancer–promoter misfolding, and lineage-specific chromatin looping defects, are largely documented in murine or bovine models, where experimental manipulation allows for high-resolution mechanistic mapping. Although these preclinical findings offer invaluable insights into potential mechanisms, caution is warranted when extrapolating them to human ART embryos, as interspecies differences in early development epigenetics and chromatin organization may influence their applicability. Clearly articulating this distinction not only strengthens the translational perspective but also highlights critical knowledge gaps that future human-focused studies should address.

### 1.4. Implications for Translation and Clinical Practice

Evidence from epigenomics, transcriptomics, and animal models indicates that ART can influence heart development by disrupting the initial regulatory mechanisms governing mesodermal lineage specification, cardiac progenitor commitment, and morphogenesis [60]. The molecular alterations occur outside the laboratory and significantly impact human health. Increasing epidemiological evidence indicates that newborns conceived using ART exhibit a higher propensity for CHDs, including atrial and ventricular septal defects, outflow tract malformations, and conotruncal anomalies [61]. Despite considering the mother’s age, infertility diagnosis, multiple gestations, and complications during pregnancy, this association remains significant. This indicates that ART procedures may directly elevate the risk of cardiovascular disease.

This clinical signal aligns with molecular-level findings from a mechanistic perspective. The ART-induced downregulation of Mef2c, Hand2, and Isl1, together with the destabilization of enhancers at the Nkx2-5 and Tbx5 loci, parallels the gene regulatory issues observed in animal models of septal anomalies and right ventricular hypoplasia [62]. The alterations in Foxh1-mediated TGFβ–Smad signaling and enhancer looping at Mef2c in ART embryos resemble those observed in loss-of-function mutants exhibiting OFT defects and conduction system delays [41]. Clinical observations indicating an elevated incidence of CHD in offspring conceived using ART should be interpreted not merely as statistical correlations, but also as phenotypic manifestations of dysregulated cardiogenic gene networks established during the preimplantation and early gastrulation stages.

Besides structural heart anomalies, children with ART may also be susceptible to cardiovascular issues that may not manifest until later in childhood or adolescence [63]. Numerous studies indicate that children and adolescents conceived via ART exhibit reduced arterial flexibility, impaired endothelial-dependent vasodilation, increased carotid intima-media thickness, accelerated pulse wave velocity, and signs of early diastolic dysfunction [64]. Alterations in the epigenome at loci regulating vascular tone, cardiac relaxation, and oxidative stress pathways, including Nos3, Sod2, Ctgf, Col3a1, and Myh6/Myh7 isoform transitions, may result in these symptoms. Significantly, these alterations align with findings from animal models, indicating that IVF induced methylation errors or chromatin remodeling defects, resulting in endothelial dysfunction, mitochondrial issues in cardiomyocytes, and modifications in fetal cardiac haemodynamics.

The placenta, a transient yet crucial organ that shares regulatory signaling pathways with the embryonic heart, appears to function as both a sensor and an effector of alterations induced by ART [65]. ART placentas frequently exhibit alterations in DNA methylation within imprinted areas (H19, Dlk1, Peg3), downregulation of angiogenic and vascular stability genes (Notch3, Dlk1, Trim28), and dysfunction of epigenetic modifiers (Dnmt3a, Ezh2, Kdm6a) [66]. The interdependent development of the placenta and circulatory system may hinder the placenta’s ability to form blood vessels, facilitate nutrient exchange, and produce hormones due to these epigenetic alterations. This may impact cardiac development by inducing hypoxia, restricting food availability, or altering hormonal signaling. Genes such as Gcm1, Pparγ, and Hand1 are recognized for their involvement in the development of both the heart and placenta. These genes may represent shared molecular targets that are disrupted by ART [67].

These findings signify a shift in the monitoring and management of ART-conceived pregnancies and offspring. Early fetal echocardiography with high-resolution Doppler and 3D sonography may enhance first-trimester screening in ART pregnancies, particularly in embryos developed through extended in vitro culture, ICSI, or those conceived by older parents. Incorporating placental biomarkers such as Plgf, sFlt1, and Papp-A, along with methylation-based assays for H19/Igf2 or Peg3, may enhance the differentiation of pregnancies at elevated risk for epigenetic and cardiovascular complications [68].

Regular cardiac monitoring of individuals conceived by ART may be essential during the neonatal and pediatric stages, despite the absence of apparent issues. Longitudinal cohort studies have identified alterations in cardiac muscle structure, elevated blood pressure, modifications in left ventricular morphology, and indications of vascular aging [69]. The findings endorse the notion that early-life cardiovascular risk assessment based on ART exposure may be beneficial, potentially through the integration of molecular indicators, such as cord blood miRNA profiles and cell-free fetal DNA methylation patterns, with phenotypic data.

Furthermore, these findings provide valuable insights that help enhance ART methodologies. Researchers are investigating methods to enhance culture conditions, including the utilization of low-oxygen environments (5% O_2_), chemically defined media devoid of serum or undefined proteins, and the incorporation of methyl donors, antioxidants, and oviductal exosomes to assess their potential in maintaining epigenetic stability [70]. Additionally, time-lapse imaging, embryo metabolomics, and transcriptome profiling of blastocoel fluid or spent media may serve as methods to assess the molecular integrity of embryos prior to transfer without direct manipulation [71].

The concept of employing molecular selection criteria for embryo transfer, encompassing not only morphology but also the stability of cardiogenic and epigenetic programs, represents a novel domain of inquiry in reproductive medicine. Screening embryos for consistent expression of Mesp1, Isl1, Gata4, and Tbx5 or for the absence of aberrant methylation at critical imprinted regions may reduce the probability of future complications. Furthermore, postnatal interventions such as incorporating methyl donors (including folate, choline, or betaine) into the diet or engaging in exercise-based vascular conditioning may present low-risk strategies to assist ART children with mild cardiovascular issues.

It is crucial to acknowledge that the application of molecular embryo profiling and biomarker-based risk classification in ART is presently constrained by various considerations. From a technological standpoint, the majority of genetic profiling techniques necessitate intrusive sampling or have yet to be standardized for routine clinical application, and their prediction accuracy in extensive, heterogeneous human populations has yet to be comprehensively confirmed. Practical factors, including cost-effectiveness, integration into current ART operations, and fair access, pose considerable obstacles. Furthermore, the ethical ramifications must be meticulously considered, encompassing the acquisition of fully informed permission, the protection of genetic and epigenetic data, and the prevention of potential exploitation of molecular information for non-medical trait selection. Mitigating these limits and ethical considerations is important prior to the responsible integration of such methodologies into conventional ART practice.

In conclusion, both clinical evidence and molecular mechanistic research increasingly indicate that ART is associated with alterations in cardiac development. The epigenetic and transcriptional instability induced by ART may result in long-term repercussions on the heart and vasculature, encompassing CHD, vascular dysfunction, and alterations in cardiac function. To enhance reproductive and cardiovascular health, it is essential to perceive the embryo not merely as a product of fertilization but also as a molecularly responsive system shaped by its cultural milieu and susceptible to transcriptional variation. To ensure the safety of ART treatments for implantation, delivery, and long-term health outcomes, it is essential to integrate basic research, developmental epigenetics, and clinical cardiology.

Table 1 provides a comprehensive overview of the essential genes and regulatory networks governing early cardiac development. It emphasizes the alteration of these genes by epigenetic factors and transcriptional dysregulation within the context of ART. Key transcription factors such as NKX2-5, GATA4, TBX5, and ISL1 initiate and enhance the specification of cardiac lineages, the division of chambers, and the architecture of conduction. Enhancer accessibility, DNA methylation, and histone changes meticulously regulate their expression. These alterations may be influenced by environmental modifications during preimplantation embryo culture. Essential epigenetic enzymes such as DNMT3A, TET3, and EZH2 modify chromatin states critical for lineage commitment. Alterations in these regulators induced by ART-related stresses, such as oxygen imbalance, culture medium constituents, or mechanical manipulation, have been associated with complications in morphogenesis, particularly concerning the formation of the right ventricle and outflow tract. Cardiac-specific microRNAs, including miR-1, miR-133, and miR-208, exhibit distinct expression patterns in embryos and placentas produced via ART. This increases the risk of transcriptional instability during cardiogenesis. The results demonstrate the vulnerability of the embryonic heart at the molecular level to epigenetic damage generated by ART, which biologically accounts for the increased incidence of congenital cardiac defects in infants conceived by ART.

## 2. Molecular Regulation of Early Cardiac Lineage Specification

### 2.1. Cardiac Transcription Factors Regulating Lineage Commitment

Cardiac specification initiates with CPCs derived from the PSM and ALPM in response to alterations in BMP2/4, FGF8, and WNT signaling pathways. A collection of transcription factors—NKX2-5, GATA4, TBX5, ISL1, MEF2C, HAND1/2, and ACTC1—collaboratively delineate cardiomyocyte fate, preserve cardiac identity, and promote chamber-specific morphogenesis [72]. These transcription factors utilize inputs from morphogen gradients to produce precise transcriptional outputs by binding to enhancers, looping chromatin, and recruiting coactivators such as p300/CBP and BRG1 [73]. This meticulously structured hierarchy is highly susceptible to epigenetic instability during in vitro cultivation, particularly under suboptimal ART conditions, such as inadequate oxygen tension, excessive FCS in the media, or fluctuations in methyl donor availability. These first alterations may disrupt the gene regulatory networks governing cardiac development, potentially leading to morphogenetic issues and increasing susceptibility to congenital heart defects.

NKX2-5 is the inaugural homeobox transcription factor exclusively located in the heart, expressed in the first heart field and the second heart field progenitors. It is essential for the development of the conduction system, ventricular trabeculation, and cardiac looping. NKX2-5 directly interacts with the promoters of MYH6, TNNT2, and ACTN2, facilitating cardiomyocyte differentiation [74]. Furthermore, it collaborates with GATA4 and TBX5 to enhance transcriptional efficacy. Knockout models demonstrate that in the absence of Nkx2-5, cardiac looping fails, atrioventricular block occurs, and the embryo succumbs by embryonic day 10.5. ART animal models demonstrate that IVF embryos exhibit reduced Nkx2-5 mRNA levels, altered nucleosome positioning at enhancer regions, and diminished H3K4me3 deposition [75]. These alterations are associated with delays in CPC commitment and reduced trabecular development, indicating that ART may epigenetically inhibit NKX2-5 by disrupting chromatin priming during zygotic genome activation.

GATA4 is crucial for myocardial differentiation. It collaborates with NKX2-5 and TBX5 to activate cardiac enhancers at the NPPA, BMP4, and Tbx20 loci. It collaborates with SMAD4 and SRF to facilitate the integration of BMP and TGFβ signals into transcription. Gata4+/− embryos had reduced cardiac muscle thickness, atrioventricular septal defects (AVSDs), and a diminished number of coronary arteries [76]. In ART models, Gata4 exhibits hypermethylation at exon 1 and reduced acetylation at its enhancer, resulting in diminished transcript activity. Researchers have successfully replicated these issues in mouse embryos under both standard atmospheric conditions and in settings deficient in methyl. A deficiency of folate in the mother, commonly observed in ART contexts, exacerbates Gata4 suppression by disrupting SAM/SAH ratios, hence altering DNA methylation processes at critical regulatory locations [77].

TBX5 regulates the identity of the left ventricle, the division of the atria and ventricles, and the development of cardiomyocytes. It binds to the T-site regions of SCN5A, IRX4, and HEY2, typically in conjunction with NKX2-5 and GATA4. The characteristics of Tbx5+/− mice include ASD, conduction delay, and hypoplastic left heart syndrome [78]. TBX5 facilitates enhancer–promoter looping in conjunction with CTCF and YY1, hence organizing chromatin domains. Blastocysts generated using assisted reproductive technology have reduced levels of Tbx5 transcripts and mislocalized chromatin insulators. Under situations that disrupt nuclear structure, such as osmotic stress or media devoid of essential amino acids/bovine serum albumin, TBX5 experiences partial silencing. This is likely due to enhancer anchors being mispositioned or the loss of transcriptional cofactors [25,79].

ISL1 delineates the SHF and regulates the influence of CPCs on the RV, OFT, and inflow tract. It integrates signals from the FGF, BMP, and WNT pathways and activates genes such as MEF2C, FGF10, and MESP1 [24]. Isl1−/− mice lack outflow tract structures, and their progenitor cells exhibit impaired growth. It is believed that ISL1+ cells possess the potential to differentiate into many cell types, including endocardial, smooth muscle, and myocardial cells. ART embryos subjected to normoxic tension (20% O_2_) have difficulties in extending the ISL1+ compartment and demonstrate hypoplasia in areas derived from the SHF [80]. These findings originate from experimental animal models; whereas direct human embryonic data are inaccessible due to ethical constraints, the involved biochemical pathways are notably conserved. Isl1 enhancers diminish H3K27ac and gain H3K27me3, whereas KDM6A/B expression decreases. These alterations indicate that issues with histone demethylation resulting from ART render the ISL1 gene less accessible, thereby leading to SHF deficiency and OFT truncation [81].

MEF2C is a MADS-box transcription factor that regulates the commitment of cardiac lineage cells and initiates the differentiation of terminal cardiomyocytes. It is positioned downstream of the TGFβ–SMAD2/3 and BMP-p38MAPK pathways, and it is essential for initiating the transcription of sarcomeric proteins [82]. Mef2c−/− embryos fail to develop the outflow tract and right ventricle, similar to the condition observed in Foxh1−/− embryos. The Mef2c anterior enhancer has a binding site for SMAD, NKX2-5, and FOXH1, which is epigenetically responsive to alterations in 5mC and H3K9ac. Embryos generated through ART exhibited diminished Mef2c levels and reduced enhancer activity, particularly when cultured in KSOM supplemented with serum [83]. Furthermore, ART embryos have reduced levels of DNMT3L and elevated levels of HDAC1, resulting in altered chromatin architecture and diminished activity of Mef2c.

HAND1 and HAND2 constitute the identities of the chambers. HAND1 is predominantly observed in the left ventricle, whereas HAND2 is primarily found in the right ventricle and outflow tract. These bHLH transcription factors regulate the development, compaction, and expression of contractile proteins in the heart [84]. Hand1−/− embryos exhibit diminished wall thickness in the LV, while Hand2−/− embryos lack an RV. Both transcription factors are influenced by hypoxic conditions, ROS, and HIF1α signaling [85]. In ART conditions characterized by elevated lactate levels or increased reactive oxygen species, Hand2 expression diminishes while miR-1 expression escalates. miR-1 is an established negative regulator. These alterations impede the functionality of RV progenitors and retard the metabolic maturation of CM.

MEF2C, GATA4, and SRF regulate the transcription of ACTC1, despite it being a structural gene. It encodes for α-cardiac actin, indicating that cardiac muscle cells have attained their terminal stage of development [86]. ART embryos, particularly those subjected to hyperosmolar stress or mechanical shear, exhibit delayed Actc1 expression and reduced affinity for the SRF enhancer. This occurs along with incomplete maturation of cardiomyocytes, diminished contractility, and disorganization of sarcomeres [87]. These findings suggest that ACTC1 serves as an indirect indicator of upstream transcription factor activity and is influenced by ART due to transcriptional noise and delayed zygotic genome activation.

These transcription factors collaborate to establish a cardiogenic gene regulatory network that is influenced by chromatin state, enhancer accessibility, and the regulation of non-coding RNA. Alterations induced by ART, such as complete methylation loss, aberrant TET and DNMT activity, or dysregulation of enhancer RNAs (including lncRNA-HANR), can dissociate these transcriptional programs from the temporal progression of embryonic development. This results in asynchronous CM differentiation, structural patterning issues, and an increased risk of congenital heart defects in infants conceived via ART.

### 2.2. Signaling Pathways That Control the Patterning of Cardiac Mesoderm

The commitment of pluripotent epiblast-derived cells to the cardiac lineage is controlled by signaling gradients that are organized in space and time and include BMP2/4, FGF8/10, WNT3A/8A, TGFβ1, NOTCH1/2, and NODAL. These morphogens control how the PSM, ALPM, FHF, and SHF are first formed and then defined. To control the fate of cardiac mesodermal cells, ligand secretion, receptor activation, intracellular cascade propagation (like SMAD, MAPK, and PI3K), and nuclear transmission of gene expression plans through chromatin remodeling all have to work together very closely [88,89]. In ART-derived embryos, however, in vitro culture circumstances often change the dynamics of ligand concentration, receptor glycosylation, and downstream phosphorylation. This changes lineage fidelity and leads to problems with heart morphogenesis.

BMP2/4 signaling through BMPR1A/B and SMAD1/5/8 increases CPC induction by turning off neural ectoderm and turning on mesendodermal gene expression, including MESP1, ISL1, and NKX2-5 [10]. High levels of BMP4 activity in the lateral mesoderm region push mesodermal progenitors toward becoming heart cells. In living organisms, different levels of BMP are needed to mark the edges of the cardiac crescent and govern HAND1/2 zonation [90]. In ART embryos, BMP4 diffusion is not normal because the matrix is too stiff or the medium does not have enough Heparan Sulphate Proteoglycans, which causes the cardiac fields to grow or move. Also, being exposed to too much oxygen raises SMAD6, a negative regulator, and causes SMAD1 to lose its phosphate group early on, which stops BMP signaling and lowers Mesp1+ lineage allocation [91].

FGF8 and FGF10 are released from the pharyngeal mesoderm and endoderm. They work through FGFR1 and 2 to turn on the RAS–RAF–MEK–ERK axis. FGF gradients are very important for the heart tube to grow from front to back, for ISL1+ SHF progenitors to multiply, and for CPCs to move toward the arterial pole [92]. Fgf10−/− mutants have underdeveloped RV and OFT, while Fgf8−/− mutants have severe looping problems and a lack of AHF. In IVF embryos, Fgf8 is epigenetically silenced because H3K9me2 accumulates at its promoter, and ERK phosphorylation does not work properly. This goes along with fewer CPCs growing and smaller SHF compartments [93]. Importantly, the fact that ART medium has fewer sulfur-containing amino acids means that FGFR sulfation is lower and receptor binding kinetics are different, which makes FGF-mediated morphogenesis even more difficult.

WNT3A/8A controls canonical β-catenin signaling through LRP6–FZD receptors. This signaling keeps pluripotency and stops the primitive streak from becoming a heart. After mesoderm induction, WNT signaling must be turned off for a certain amount of time in order to specify CPC [94,95]. DKK1 and CER1 are secreted WNT inhibitors that allow for GATA4 and MEF2C activation, which allows for cardiac gene derepression to happen. ART embryos often do not stop WNT3A from working, which keeps β-catenin in the nucleus, T expression going, and CPC emergence going wrong. Also, higher levels of SNAIL and ZEB1, which are WNT-responsive EMT drivers, can slow down the epithelial–mesenchymal transitions that are important for forming the heart tube [96].

TGFβ1 controls the expression of Foxh1, Mef2c, and Nkx2-5 in the AHF through TGFBR2/ALK5 and downstream SMAD2/3. It is also important for endocardial cells to undergo EMT during valve development. In ART embryos, high or low levels of TGFβ1 have been related to delayed ZGA and problems with Foxh1 moving to the nucleus. This makes it harder for the Mef2c enhancer to work and changes how the OFT forms. Oxidative stress caused by ART raises SMAD7, which is a feedback inhibitor. This causes canonical TGFβ targets to be downregulated and SHF-derived lineages to be misregulated [97,98].

When JAG1/2 and DLL1/4 turn on NOTCH1/2, they work through RBPJκ to stop CPC growth, encourage trabeculation, and control the creation of valves. NOTCH1 stops BMP2 from working and helps the endocardial identity form in the early embryo. ART techniques that involve long-term in vitro culture change the strength of NOTCH signals by causing NUMB to be upregulated too soon, which is an intracellular NOTCH inhibitor [99]. Because of this, the creation of endocardial cushions and the specification of valve progenitors are not working properly. Also, ART embryos have abnormal RBPJ binding at Hey1/2 enhancers, which makes trabeculation happen more slowly or not at all.

Lastly, NODAL, a member of the TGFβ family, is very important for creating L–R asymmetry and shattering embryonic symmetry by turning on LEFTY2 and PITX2. If Nodal signaling is blocked or lost, the heart’s looping becomes random. ART embryos have lower levels of Nodal expression. This could be because its enhancer (ASE) is methylated or Foxh1, its nuclear effector, is not working properly [100]. These results are in line with greater incidences of situs abnormalities, D-looping malformations, and double outlet right ventricle in animal models that were conceived by ART.

In short, ART causes molecular instability during the preimplantation phase, which changes ligand secretion, receptor function, and the way the primary cardiogenic pathways work inside cells. These problems make it harder for precise morphogenetic events to happen, like CPC induction, field specification, looping, and OFT development. This raises the chance of structural heart defects in embryos that come from ART.

### 2.3. Epigenetic Influences on the Activation of Cardiogenic Genes

Epigenetic control serves as a crucial biological connection between environmental stimuli and gene expression during embryonic development. It regulates the temporal and spatial aspects of transcriptional processes essential for mesodermal lineage specification, chamber formation, and morphogenetic remodeling throughout cardiac development. These mechanisms, encompassing DNA methylation, histone PTMs, chromatin remodeling, and imprinting, establish transcriptional competence at critical cardiac loci such as Nkx2-5, Mesp1, Tbx5, Gata4, Isl1, and Mef2c. Emerging data indicates that epigenetic instability associated with ART, particularly IVF and in vitro culture, affects cardiogenic gene networks, hence increasing the risk of CHDs.

DNA methylation constitutes one of the initial and most enduring alterations to the epigenome that occur during embryonic reprogramming. During initial lineage commitment, the de novo methyltransferases Dnmt3a and Dnmt3b introduce 5-methylcytosine (5mC) [101]. Following replication, Dnmt1 retains these modifications. During cardiac mesoderm specification, loci such as Nkx2-5, Mesp1, and Isl1 undergo selective demethylation to initiate transcription. Embryos produced using assisted reproductive technology frequently exhibit atypical methylation patterns due to environmental alterations during the zygotic genome activation period [102]. Embryos cultivated in oxygen-rich or serum-abundant conditions exhibit hypermethylation of the Nkx2-5 enhancer and Mesp1 promoter. Furthermore, ART animals frequently have diminished levels of Tet1/2/3, enzymes that synthesize 5-hydroxymethylcytosine (5hmC) and facilitate active demethylation [103]. This complicates the activation of critical cardiogenic loci. This anomalous methylation state impedes the emergence of CPC and disrupts the patterning of the FHF and SHF compartments that follow.

Histone post-translational modifications are equally crucial for regulating chromatin accessibility and the binding of transcription factors throughout cardiac development. The incorporation of three methyl groups into histone H3 lysine 4 (H3K4me3) and the attachment of an acetyl group to H3 lysine 27 (H3K27ac) are both substantially associated with gene activation. Suv39h1/2 and Ezh2 introduce two methyl groups to H3K9me2 and H3K27me3, therefore inhibiting transcription. During the initial stages of cardiac development, the Gata4, Tbx5, and Hand1 loci exhibit significant levels of H3K4me3, indicating their preparedness for activation. However, ART-induced stress, particularly oxidative stress and abnormal metabolite concentrations (such as reduced α-ketoglutarate or S-adenosylmethionine), disrupts the function of histone methyltransferases and demethylases, altering these modifications. Researchers using mouse IVF embryos have demonstrated that excessive H3K9me2 and insufficient H3K27ac on the Mef2c and Tbx1 promoters inhibit the timely initiation. Whether these specific histone mark alterations occur in human ART embryos remains to be confirmed [104].

ATP-dependent complexes such as SWI/SNF, which include Brg1 (encoded by Smarca4) and Baf60c, regulate chromatin remodeling. These remodelers reposition nucleosomes to facilitate easier access for transcription factors such as Nkx2-5, Gata4, and Mef2c. Brg1 collaborates directly with Nkx2-5 to regulate enhancers that govern the development of the conduction system and the specification of the ventricles [105]. Research employing ATAC-seq on cultured blastocysts indicates that assisted reproductive technology environments diminish the levels of Brg1 mRNA and protein. They further demonstrate that essential cardiac enhancers exhibit reduced accessibility within chromatin. This impaired remodeling landscape diminishes the efficacy of the transcriptional machinery and results in the heart muscle becoming insufficiently developed in subsequent stages.

The epigenetic regulation of imprinted loci is significantly diminished in ART embryos because of alterations in the processes of methylation erasure and re-establishment during culture. Genes such as Igf2, H19, Dlk1, Peg3, and Kcnq1ot1 are recognized for their influence on cardiac development, metabolism, and cellular equilibrium. For instance, Igf2 is crucial for the growth and hypertrophy of the cardiac walls during ventricular development. The loss of imprinting or biallelic repression of Igf2 in ART embryos has been associated with diminished ventricular mass and septal abnormalities [106,107]. Comparable results for Kcnq1ot1, which regulates the Cdkn1c–Kcnq1 cluster, suggest that imprinting errors are responsible for the delayed ventricular development and arrhythmogenic characteristics.

The many epigenetic alterations in ART-derived embryos result in a cardiac genome that is either non-transcribable or transcribed at inappropriate times. Aberrant promoter methylation, histone signature abnormalities, chromatin compaction defects, and imprinting errors collectively inhibit the initiation and progression of cardiogenic transcriptional waves. These alterations are not only permanent; they may evolve over time, influencing both embryonic heart development and the long-term health of the cardiovascular system in individuals conceived by ART. Comprehensive understanding of the epigenetic landscape of cardiogenesis in the context of ART requires further research that integrates epigenomic profiling, such as ChIP-seq and WGBS, with cardiac phenotyping to develop targeted therapeutics for epigenetic rescue.

### 2.4. Non-Coding RNAs in Initial Cardiac Development

Non-coding RNAs (ncRNAs), including microRNAs (miRNAs), long non-coding RNAs (lncRNAs), and circular RNAs (circRNAs), play a crucial role in early cardiac development. These chemicals influence lineage determination and morphogenetic processes by altering chromatin configurations, mRNA stability, and protein synthesis. They also refine transcriptional and epigenetic mechanisms. In the meticulously orchestrated process of cardiogenesis, ncRNAs facilitate the identification, development, and specialization of CPCs [108]. Recent research indicates that ART, particularly IVF, may significantly alter the expression patterns of cardiogenic ncRNAs. This is likely due to epigenetic drift resulting from adverse cultural contexts, oxidative stress, or alterations in metabolic inputs.

The miR-1/133 cluster comprises some of the most ancient and well-conserved cardiac regulatory miRNAs. These miRNAs originate from bicistronic units and are essential for regulating the growth rate of CPCs, preventing both excessive and insufficient proliferation. miR-1 facilitates cellular differentiation by inhibiting Hdac4, hence preventing Mef2c from functioning. This facilitates the transcription of sarcomeric genes such as Myh6 and Tnnt2 [109]. Conversely, miR-133 sustains the proliferation of progenitor cells by targeting Srf, Ctgf, and Cyclin D2. Alterations in the miR-1 to miR-133 ratio result in disorganized sarcomere assembly and induce looping complications. ART-derived mouse embryos exhibited delayed production of miR-1-2 and hypermethylation at its promoter, potentially due to alterations in Dnmt3a activity during the transition from the four-cell stage to the morula stage [110]. This alteration disrupts the timing of CPC specification and results in mispatterned chambers.

The miR-1792 cluster, also known as oncomiR-1, is a significant element in early cardiac development, particularly within the SHF. Inhibiting pro-apoptotic genes such as Bim, Pten, and Tgfbr2 facilitates the growth and longevity of Isl1+ CPCs. The absence of this cluster in mouse embryos results in outflow tract hypoplasia and ventricular septal defects, akin to secondary heart field dysfunction [111]. IVF conditions are associated with reduced expression levels of miR-17 and miR-19b, which correlate with premature exit from the CPC pool and increased apoptotic signaling. The alterations are likely attributable to the diminished accessibility of the miR-17~92 promoter’s chromatin, as blastocysts exhibit reduced levels of H3K4me3.

miR-208a/b and miR-499 are intronic microRNAs located inside the Myh6/Myh7 and Myh7b loci, respectively. They alter the switching of myosin isoforms and contribute to the determination of the ventricular chamber’s identity. These miRNAs inhibit transcriptional repressors such as Sox6, Sp3, and Thrap1, facilitating the maturation of cardiac muscle [112]. ART disrupts these miRNAs, particularly because of irregularities in histone modifications at the enhancers of their host genes. This results in ventricular hypoplasia and abnormal septation. Animal studies indicate that oxidative stress induced by culture alters the levels of Brg1 and Hdac1, which are crucial for modifying chromatin structure at these locations.

lncRNAs are increasingly recognized as equally significant as other RNA types in regulating cardiac growth. Braveheart (Bvht) is a long non-coding RNA that exclusively influences cardiomyocytes. It engages with Swi/Snf and PRC2 complexes to modify the chromatin landscapes of Mesp1, Nkx2-5, and Gata4, thereby priming mesodermal progenitors for their cardiac destiny. When Bvht is disrupted, the transition from mesoderm to CPC does not occur. Elevated glucose, serum supplementation, or oxidative stress in ART culture conditions have been shown to suppress Bvht by increasing repressive H3K27me3 marks at its promoter. Fendrr is an lncRNA that regulates cardiogenic transcription factors Foxf1 and Pitx2 via modifying histones. When Fendrr is absent, the left–right patterning and posterior cardiac field expansion are ineffective. These elements are also demonstrated in ART-derived animal models [113].

Kcnq1ot1, a long non-coding RNA inside the imprinted Kcnq1 gene, regulates cardiac development and conduction. It functions by recruiting G9a and PRC2 to silence adjacent genes such as Cdkn1c, which regulates cardiomyocyte development. Researchers have discovered that ART embryos exhibit a loss of imprinting at Kcnq1ot1. This may elucidate why ART-conceived progeny exhibit enduring alterations in heart rate variability and myocardial contractility [114,115].

Circular RNAs (circRNAs), formed through back-splicing of exons, play a crucial role in cardiac development. Circ-Sirt1 sequesters miR-93, hence enhancing Mef2c expression and promoting cardiomyocyte survival under oxidative stress. Factors influencing circRNA biogenesis, such as the disruption of Qki and Fus, RNA-binding proteins crucial for circRNA maturation, result in significant alterations in the circRNA landscape. This may further destabilize miRNA–mRNA networks critical for cardiac development [116].

The ncRNAs in ART-derived embryos are disrupted during multiple stages of development. Alterations in miRNA and lncRNA profiles may create an epigenetic imprint that persists beyond the blastocyst stage and influences cardiac function postnatally. Research utilizing transcriptome and short RNA sequencing on ART-derived cardiomyocytes indicates that miR-1, miR-499, and Bvht remain repressed, whereas stress-responsive lncRNAs such as Neat1 and Malat1 are activated. Both lncRNAs are associated with fibrosis and alterations in cardiac function.

In summary, ncRNAs constitute a sophisticated layer of regulatory networks that link signaling pathways with chromatin remodeling and transcriptional outcomes during the initial phases of cardiac development. ART procedures disrupt the expression and function of these ncRNAs by altering the metabolic signals and epigenetic landscape of the preimplantation embryo. Such issues render the CPC unstable, induce chamber irregularities, and may ultimately result in chronic heart disease. Comprehending these ncRNA-mediated regulatory networks in ART contexts may facilitate the identification of diagnostic biomarkers and early interventions that mitigate the risk of CHD in individuals created via ART.

Table 2 shows the important regulatory roles of several non-coding RNAs in heart specification, such as their molecular targets and how they affect gene expression in the heart. It also explains how ART treatments, especially IVF and embryo culture, might change these pathways by changing chromatin dynamics, epigenetic reprogramming, and oxidative stress. These kinds of problems may be the cause of heart problems in ART-derived children and could lead to new epigenetic and transcriptomic biomarkers for figuring out who is at risk for heart disease early on.

## 3. ART and Congenital Heart Disease—Clinical Correlates and Mechanistic Insights

### 3.1. Epidemiological Correlation Between ART and Congenital Heart Disease

The utilization of ART, including IVF and ICSI, has increased globally. This has assisted millions of couples in conceiving children. As ART has gained popularity, an increasing number of individuals are scrutinizing its potential developmental impacts, particularly with congenital defects. CHD is the most prevalent and clinically significant anomaly among these conditions. This necessitates further investigation into the frequency, underlying patterns, and molecular mechanisms involved in ART-conceived children.

Extensive epidemiological research globally indicates that children conceived through ART have a higher likelihood of developing CHD compared to those conceived naturally. The SMART Collaborative conducted a comprehensive study analysing data from over 4.6 million live births in Florida, Massachusetts, and Michigan. They determined that 58.6 per 10,000 live births were conceived using ART, in contrast to 47.5 per 10,000 live births that were not ART-conceived. After including the mother’s age, multiple pregnancies, and socioeconomic characteristics, the disparity persisted, yielding an adjusted risk ratio (aRR) of 1.28. Notably, singleton ART babies, typically perceived as having a reduced risk compared to multiples, exhibited an elevated risk (aRR 1.38). This indicates that the increased likelihood of CHD is not only attributable to twinning, premature birth, or complications related to shared placental perfusion [5,117].

Additionally, several cardiac issues have been observed with greater frequency in ART populations compared to the general populace. These encompass ASDs, VSDs, conotruncal defects (such as tetralogy of Fallot), and transposition of the great arteries [118]. Certain research has indicated that ART elevates the likelihood of heterotaxy syndromes and laterality problems. These issues stem from underlying complications in embryonic development, such as difficulties in developing the left–right axis. These findings suggest that ART influences critical early developmental processes, such as mesodermal patterning and the differentiation of CPC lineages, rather than only impacting organ growth at later stages of gestation [119].

There have also been associations specific to various processes. Assisted hatching, a technique that compromises the zona pellucida prior to embryo transfer, has been associated with an increased incidence of congenital heart defects, particularly in singleton births. Fresh embryo transfers appear to carry greater risks compared to frozen–thawed cycles. This may occur due to hormonal overstimulation of the endometrium or the presence of oxidative stress during in vitro culture. Maternal ovulatory disorders, such as PCOS, which frequently necessitate ART, are associated with an elevated baseline risk of heart anomalies in offspring. This exacerbates the risks of the operation and demonstrates that ART-related congenital heart disease has several aetiologies [5,120].

These epidemiological findings are significant not only from a statistical perspective but also for clinical and translational research. Individuals with congenital heart disease and healthcare systems must manage it indefinitely. This typically entails prompt surgical intervention, prolonged pharmacological support, and extended neurodevelopmental surveillance. At the population level, the overall risk elevation attributable to ART is modest; yet, it results in thousands of additional cases of CHD annually. This underscores the necessity of comprehending the mechanisms of ART and devising strategies to mitigate it.

Moreover, conventional registries that focus only on prenatal diagnoses may not capture the complete spectrum of heart complications associated with ART. Longitudinal studies employing fetal echocardiography, high-resolution imaging, and genetic screening are beginning to identify minor issues and subclinical cardiac conditions, such as conduction system abnormalities and diastolic dysfunction. Initial issues with gene expression, chromatin architecture, or epigenetic imprinting during preimplantation development may account for these anomalies [121]. Epidemiology provides fundamental connections while also enabling molecular examination. The subsequent sections will examine the significance of transcription factors, non-coding RNAs, and epigenetic modulators—many of which are highly responsive to environmental stressors associated with ART—in influencing cardiac development in this particular patient cohort.

### 3.2. ART and Issues with Left–Right Patterning

The left–right (L–R) axis must be established during early development to ascertain the proper spatial orientation of internal organs, particularly the heart, which is the first functional organ to exhibit asymmetry [122]. Molecular signals, ciliary movement, epigenetic factors, and transcriptional networks must collaborate within a brief timeframe following gastrulation. Issues with left–right patterning can lead to significant congenital heart disease CHD manifestations, including dextrocardia, situs inversus, heterotaxy, and atrioventricular canal defects. Recent data indicates that ART, particularly IVF and in vitro culture techniques, may disrupt this crucial developmental process by altering gene expression and epigenetic programming during the initial stages of embryonic development [123].

The initial molecular event establishing the left–right axis in mammalian embryos occurs at the embryonic node. The movement of monocilia on nodal cells induces a fluid flow directed to the left. Stationary mechanosensory cilia can detect this flow, resulting in the activation of signaling pathways in an asymmetrical manner. Nodal, Lefty1/2, and Pitx2 constitute the fundamental signaling cascade. They are exclusively activated on the left side of the LPM. Nodal signaling, regulated by a feedback loop involving Lefty antagonists and Cerl2, induces the expression of Pitx2, a transcription factor that governs the left-sided development of organs, including cardiac looping. Errors in this symmetry-breaking mechanism can significantly impact the heart at an early stage and in a highly sensitive manner. Alterations in Nodal–Pitx2 signaling can result in the heart being mispositioned and the chambers being misaligned [124].

Numerous mechanisms exist by which ART treatments may alter the L–R patterning at the molecular level. In vitro fertilization and embryo culture generally expose preimplantation embryos to settings characterized by varying redox states, diminished nutrient levels, and elevated oxygen concentrations, which may influence ciliary growth and function [125]. Oxidative stress impedes the growth and movement of nodal cilia, hence complicating the establishment or detection of leftward flow. Numerous studies using mouse models have shown that embryos cultured in vitro exhibit randomization or reversal of cardiac looping and misexpression of left–right markers, including Nodal and Pitx2. The findings in murine embryos provide valuable insights into putative pathways in human ART-conceived embryos, but should be considered translational rather than direct clinical proof [126].

Epigenetic dysregulation is a significant mechanism by which ART may disrupt L–R patterning. Early exposure of embryos to culture conditions can alter the patterns of histone modification and DNA methylation in critical regulatory regions of L–R genes. H3K27me3-mediated repression regulates the Pitx2 promoter and its enhancer regions. This inhibition is dynamically eradicated to facilitate left-specific expression [127]. Alterations in the activity of chromatin modifiers such as EZH2 and KDM6A/B induced by ART can inhibit or attenuate Pitx2 expression, thereby disrupting or reversing cardiac looping. The equilibrium between Nodal activation and Lefty repression is regulated by the contacts between enhancers and promoters, influenced by chromatin structure. This structure is particularly susceptible to environmental stress during preimplantation development.

Furthermore, ART has been shown to alter the expression of maternal-effect genes and lncRNAs that regulate left–right symmetry. Superovulation techniques or culture media can disrupt genes such as Zar1, Mater, and Nlrp5, which are crucial for cytoplasmic programming of oocytes and early activation of the zygotic genome. These maternal regulators modify the epigenetic configuration of early embryonic chromatin and influence the expression of L–R patterning genes via trans-acting pathways. In ART embryos, manipulating these upstream regulators can alter the spatial identity of embryonic axes prior to the occurrence of nodal flow.

These mechanistic findings are corroborated by clinical evidence. Epidemiological studies indicate that infants conceived via ART are at an increased risk of developing laterality defects, including situs ambiguus, right atrial isomerism, and transposition of the great arteries. Such issues are infrequent in the general populace but are far more prevalent in ART cohorts, particularly those involving fresh embryo transfers or prolonged in vitro incubation until the blastocyst stage [128]. These issues frequently occur together with congenital heart defects that affect septation, outflow tract alignment, and venous return routes. This illustrates the significance of L–R disruption in the formation of complex cardiac anomalies.

Ultimately, we must consider the impact of ART on L–R asymmetry within the broader context of gene–environment interactions. An intricate exposome that engages with the embryo’s genetic and epigenetic framework comprises variations in culture material, embryo handling, oxygen levels, and endometrial receptivity. The investigation of single-cell transcriptomics and methylomes in ART-derived embryos will enhance our understanding of the enduring effects of transient environmental variables on cardiac development, particularly regarding axis specification.

In summary, ART therapies provide a significant environmental variable that can alter the first mechanisms responsible for breaking symmetry throughout embryogenesis. ART may contribute to the emergence of congenital cardiac defects that influence laterality by affecting ciliary function, transcriptional regulation, and epigenetic modification. Understanding these molecular vulnerabilities is crucial not only for risk assessment and prenatal diagnosis but also for optimizing ART techniques to safeguard early embryonic patterning.

### 3.3. Halting the Progression of the Second Heart Field and Outflow Tract

The SHF is a transient, multipotent collection of cardiac progenitor cells located in the pharyngeal mesoderm, positioned anteriorly and laterally to the primary heart field. The elongation of the heart tube is crucial for the development of the RV, the OFT, and portions of the atria. The correct alignment of the principal arteries and the division of the outflow tract into the aorta and pulmonary artery rely on adequate second heart field development [92]. Issues with SHF deployment or differentiation are closely associated with conotruncal anomalies such as chronic truncus arteriosus, DORV, transposition of the great arteries (TGA), and TOF. These represent several forms of CHDs.

A hierarchical network of transcription factors and signaling molecules regulates SHF progenitors at the molecular level. ISL1 is a LIM–homeodomain transcription factor integral to SHF identity and essential for the proliferation and migration of progenitor cells [129]. MEF2C is a MADS-box transcription factor that functions subsequent to ISL1 to facilitate cardiomyogenic differentiation and cardiac hypertrophy in the outflow tract. TBX1 is a T-box gene associated with DiGeorge syndrome (22q11.2 deletion) [130]. The development of the heart from the pharynx and the SHF is essential, particularly for the patterning of the aortic arch. Additional regulators, such as FOXC1/2, NKX2-5, and GATA4, prevent progenitor cells from premature differentiation and assimilate signals from the pharyngeal endoderm and neural crest.

The SHF transcriptional network interacts with significant morphogenetic pathways, including FGF (notably, FGF8 and FGF10), BMP, Wnt/β-catenin, Notch, and TGF-β. These signals alter the timing of the cell cycle, lineage specification, and the transition from EMT in SHF cells. For example, FGF8 derived from the pharyngeal ectoderm influences SHF progenitors to proliferate and maintain a reservoir of undifferentiated cells [92]. Conversely, BMP and TGF-β signaling at specific spatiotemporal thresholds facilitates the differentiation of cardiac muscle cells. A Smad-responsive enhancer featuring a composite binding site for FOXF1, NKX2-5, and FOXH1 regulates MEF2C expression. This suggests a significant connection between TGF-β signaling and cardiac transcriptional commitment.

IVF and extended embryo culture are instances of ART that may disrupt SHF development at several levels. Preimplantation embryos cultivated in suboptimal conditions exhibit increased oxidative stress, nutritional deficiencies, and alterations in osmolarity. All of these factors can impede the ability of developing progenitors to transmit signals and regulate transcription. Research on murine embryos indicates that blastocysts generated via in vitro fertilization exhibited reduced levels of Isl1, Mef2c, and Tbx1. They also possess disordered pharyngeal mesoderm and abbreviated OFTs. In numerous instances, these embryos exhibit a deformed right ventricle, a diminutive conus arteriosus, and an issue with aortopulmonary septation.

From a chromatin perspective, ART may induce significant alterations in the epigenetics of SHF-specific loci. Environmental signals significantly influence DNA methylation and histone modifications at the regulatory regions of Mef2c, Tbx1, and Fgf8. Embryos treated to serum-containing media exhibited elevated levels of histone H3K27me3 at the Mef2c enhancer, correlating with less transcriptional activity [131]. Conversely, global DNA hypomethylation induced by ART may activate genes that are typically inactive in the SHF, potentially resulting in ectopic lineage choices. Transcriptomic and ChIP-seq analyses of murine embryos exposed to ART and hESCs developing into cardiogenic cells corroborate these findings.

Moreover, maternal and zygotic determinants influencing early chromatin accessibility, including DPPA3, ZFP57, KDM6B, and DNMT3A/B, are impacted by epigenetic drift caused by ART. These chromatin remodelers influence the activation of SHF genes by establishing TADs and enhancer–promoter looping connections [132]. Interfering with these epigenetic regulators during ART may initiate the SHF program at varying periods or prevent its initiation altogether, hence complicating the growth of the heart tube and the proper formation of the outflow tract.

Numerous epidemiological studies indicate that infants conceived via ART exhibit a higher propensity for developing conotruncal cardiac anomalies. These encompass issues with the aortic arch, truncus arteriosus, and pulmonary valve stenosis, which are prevalent complications arising from SHF failure. The association is strongest in ART cycles employing fresh embryo transfers and culture extending to the blastocyst stage. This discovery suggests that later-stage embryos may exhibit greater epigenetic instability when subjected to prolonged exposure to artificial media and oxidative stress. Prenatal echocardiography in ART pregnancies occasionally detects a reduced OFT length and atypical orientation of the main arteries as early as 13 to 15 weeks of gestation.

Furthermore, human in vitro models that replicate ART conditions, such as high-glucose or high-lactate media, induce differentiation in hESCs and iPSCs that deviates from typical first heart field fate, and SHF-like populations exhibit suboptimal growth rates. Analysis of the transcriptome of these cells reveals a downregulation of ISL1, TBX1, and FOXC2, indicating that SHF programming is responsive to environmental alterations.

In conclusion, the SHF represents a very intricate and sensitive domain of early cardiac development. ART approaches can alter the destiny and function of SHF progenitors by disrupting the equilibrium of redox processes, transcriptional signaling, and epigenetic regulation. This may result in various conotruncal anomalies. In the future, researchers should investigate how ART alters enhancers in SHF cells, examine the interaction between maternal and zygotic epigenetic variables, and design improved culture techniques that preserve the developmental capacity of this crucial cardiac region.

### 3.4. Modifications to the Epigenome Within Cardiac Transcriptional Networks

Transcriptional programs that govern heart development are meticulously regulated, although their timing and precision rely on a complex epigenetic environment that alters chromatin shape, hence modulating gene accessibility. This epigenetic system encompasses DNA methylation, post-translational histone modifications, nucleosome positioning, chromatin remodeling complexes, and non-coding RNAs, including microRNAs and long non-coding RNAs [133]. These systems collaborate to ensure the precise expression of cardiac-specific genes at the appropriate time and location, facilitating a seamless transition from mesodermal progenitors to differentiated cardiomyocytes. The regulatory layers are crucial throughout the initial phases of heart development, particularly between gastrulation and the formation of the linear heart tube.

The dynamic regulation of cardiac master genes such as NKX2-5, TBX5, GATA4, ISL1, MEF2C, HAND1, and HAND2 is a critical aspect of cardiogenesis. These transcription factors bind to cardiac enhancers and promoters, initiating a cascade of gene expression essential for chamber specification, myocardial proliferation, conduction system development, and valvulogenesis [134]. The chromatin environment surrounding them significantly influences their functionality. To effectively recruit transcription factors and coactivators, enhancers must possess active histone modifications such as H3K4me1 and H3K27ac. Conversely, poised or repressed chromatin marked by H3K27me3 or H3K9me3 impedes transcriptional activity. Polycomb repressive complexes, such as PRC2, and Trithorax group proteins maintain this balance in embryonic stem cells and cardiac progenitors. This enables the cells to rapidly activate or deactivate based on the signals received [135].

ART, particularly IVF, disrupts this fragile epigenetic equilibrium. During the initial stages of implantation, the embryo undergoes significant epigenetic modifications. For instance, global demethylation occurs alongside the lineage-specific re-establishment of de novo methylation. Interventions related to ART, including exposure to suboptimal culture media, oxidative stress, external hormones, and alterations in nutritional composition, can diminish the efficacy of this therapy [136]. Research in mice has shown that IVF embryos exhibit aberrant methylation at the promoters of NKX2-5, GATA4, and HAND2. This results in transcriptional attenuation and complications in cardiac patterning. Furthermore, chromatin accessibility at enhancers regulating MEF2C and ISL1 is diminished under ART-mimicking culture conditions, impeding the activation of subsequent cardiogenic pathways.

Additional molecular investigations indicate that ART inhibits the functionality of critical chromatin remodeling complexes. Activating cardiac enhancers requires BRG1 (a component of the SWI/SNF complex), CHD4 (a nucleosome remodeler associated with the NuRD complex), and histone demethylases such as KDM6A and KDM6B. IVF embryos exhibit differential localization or expression of chromatin regulators inside the nucleus, resulting in improper spacing of nucleosomes and a reduction in histone acetylation marks [137]. These alterations to the epigenome impede enhancer–promoter looping, hence decelerating transcriptional elongation and hindering the functionality of cardiac RNA polymerase II machinery.

Scientists are concerned that DNA methylation is abnormal in ICRs in embryos produced by ART. This may impact the entire body, influencing cardiac development. Losing imprinting at the IGF2-H19, KCNQ1OT1-CDKN1C, or GRB10 loci is associated with abnormal fetal development and alterations in cardiac morphology [106]. GF2 is crucial for the proliferation of cardiac myocytes and the hypertrophy of ventricular walls. Epigenetic instability in ART embryos may result in dysregulated IGF2 expression, potentially leading to ventricular septal defects or right ventricular outflow tract deformities. These are prevalent in epidemiological research, including children using ART.

The patterns of histone alterations in ART embryos differ from those in conventionally created embryos. IVF embryos exhibit reduced levels of the activation marks H3K27ac and H3K4me3 at cardiac-specific gene promoters, and increased levels of the repressive mark H3K27me3 [138]. This histone-mediated repression impedes the activation of critical genes such as TBX5 and MEF2C, hence hindering or halting the process of chamber morphogenesis. Bivalent domains, characterized by the presence of both H3K4me3 and H3K27me3, often transition to active marks during differentiation; nevertheless, this transition may not occur, thereby hindering development or resulting in improper cardiomyocyte formation [139].

Non-coding RNAs are crucial for maintaining the heart’s identity and preventing the emergence of alternative lineages. miR-1, miR-133, and miR-208 are crucial cardiac microRNAs that facilitate sarcomere assembly, inhibit non-cardiac transcriptional programs, and regulate ion channel synthesis [140]. Research has demonstrated that ART techniques diminish the amounts of these microRNAs, resulting in issues with cardiomyocyte growth and electrical instability. For instance, miR-1 inhibits HAND2, hence regulating ventricular formation. A reduction in miR-1 may lead to myocardial hyperplasia or arrhythmogenic substrates. Similarly, miR-133 regulates the proliferation and apoptosis of cardiac progenitors [141]. Disturbance may result in anatomical complications, such as thin-walled ventricles.

Recent research integrating transcriptomics, epigenomics, and chromatin accessibility techniques, such as ATAC-seq and ChIP-seq, has demonstrated that early epigenetic misprogramming induced by ART has enduring effects on cardiac lineage trajectories [142]. Differentiated cardiomyocytes derived from ART embryos have altered enhancer repertoires, misconfigured enhancer–promoter pairs, and enduring epimutations in genes such as ISL1 and TBX1. The genetic alterations are associated with physical characteristics such as ASDs, improper looping morphogenesis, and ventricular trabeculation issues.

Ultimately, alterations in the epigenetic framework of cardiac transcriptional networks link environmental modifications to congenital heart disease at the molecular level. Modifications induced by ART, including alterations in methylation, histone modification, chromatin remodeling, or non-coding RNA synthesis, can destabilize the cardiac gene regulatory network at multiple levels. These epigenetic alterations elucidate the increased incidence of congenital heart anomalies in children conceived with ART and the underlying mechanisms of these changes. An enhanced understanding of these molecular pathways may advance ART methodologies and provide researchers with novel strategies for addressing or preventing issues in at-risk embryos.

Table 3 illustrates that an increasing body of data indicates the critical role of epigenetic regulators in ensuring the proper progression of cardiogenesis. Enzymes such as DNMT1 and DNMT3A/3B are responsible for maintaining and establishing DNA methylation patterns that safeguard cardiac lineage commitment. EZH2 and KDM6A/KDM6B are histone modifiers that alter the levels of H3K27me3 at cardiac enhancers and promoters, hence affecting chromatin accessibility. Chromatin remodeling complexes, particularly BRG1 (SWI/SNF) and CHD4 (NuRD), facilitate the repositioning of nucleosomes, thereby enabling transcription factors to engage with cardiac regulatory elements. Cardiac-specific microRNAs, including miR-1, miR-133, and miR-208, regulate gene expression by inhibiting the formation of alternative lineages and maintaining the integrity of sarcomeres and conduction pathways.

Assisted reproductive technologies disrupt epigenetic processes in various ways, resulting in prolonged or more challenging activation of cardiac transcriptional networks. This disturbance manifests as diminished histone acetylation, restrictive chromatin profiles at critical loci (including NKX2-5, TBX5, and HAND2), and reduced synthesis of cardiogenic microRNAs. Molecular alterations of this nature may increase the likelihood of structural cardiac anomalies and issues with the proper development of cardiac muscle in embryos created by ART. This underscores the significance of considering epigenetic health during the utilization of ART.

## 4. Clinical Implications and Translational Perspectives

### 4.1. ART-Induced Cardiovascular Risk: Emerging Clinical Evidence

Over the past 20 years, more and more epidemiological evidence has made many worried about the long-term health of people who were conceived via ART. ART has changed the way infertility is treated and led to the birth of more than ten million children around the world. However, multiple cohort studies and registry-based analyses have found that ART-conceived neonates are more likely to have CHDs, with estimates of the risk being 1.3 to 1.5 times higher than that of naturally conceived controls. Atrial and ventricular septal defects, conotruncal malformations (such as Tetralogy of Fallot and transposition of the great arteries), and outflow tract abnormalities are some of the most common types of birth defects. These results seem to be the same for several ART methods, such as traditional IVF, ICSI, and frozen embryo transfer. This suggests that the danger is not confined to only one method [128].

There are many variables that contribute to these cardiovascular risks, including both parental and iatrogenic factors. On the parental side, being older, overweight, or having diabetes, as well as having disorders that make it hard to get pregnant (such as polycystic ovarian syndrome or endometriosis), may make gametes less healthy and cause early embryonic epigenetic instability [143]. On the ART side, important times for handling embryos, such as superovulation, in vitro culture, zona modification, and cryopreservation, could all mess up the precisely controlled epigenetic programming that controls heart development. For example, changes in oxygen tension or exposure to non-physiological media components can change the redox balance, cause stress in mitochondria, and lead to abnormal DNA methylation in cardiogenic loci [144].

Along with congenital defects, more and more long-term studies are finding symptoms of subclinical cardiovascular disease in children, teens, and young adults who were conceived through ART. Some of these are higher systolic and diastolic blood pressure, thicker carotid intima-media, less flow-mediated dilatation, and early symptoms of stiff arteries. It is interesting that some of these changes happen even when there are no clear signs of CHD [145]. This suggests that ART may have long-lasting impacts on regulating vascular tone, making endothelial cells more sensitive, or maturing cardiomyocytes through modest epigenetic or metabolic imprinting.

At the molecular level, ART-related cardiovascular abnormalities may come from the wrong regulation of important cardiac developmental genes (such as NKX2.5, GATA4, HAND1/2) because enhancer methylation or histone modification went wrong during early lineage commitment [146]. Also, epigenetic suppression or overexpression of microRNAs, including miR-1, miR-133, and miR-208, which are important for cardiomyocyte differentiation, maturation of the conduction system, and response to oxidative stress, could lead to delayed cardiac patterning or functional immaturity [147]. Animal models reinforce these ideas by showing that ART and in vitro culture change the structure of the heart muscle and the way cardiogenic transcription factors are expressed across time and space, even when there are no obvious abnormalities.

All of this information shows that ART offspring need to be more conscious of their cardiovascular health and have long-term follow-up. Even though the risk of severe CHDs is still minimal, the fact that subtle structural and functional changes are consistently found suggests that ART may cause a range of cardiometabolic programming outcomes, from changes that do not show up in clinical tests to full-blown illness. Finding the molecular mediators of these effects could help with early risk assessment, prevention, and maybe even the modification of ART regimens to lessen these long-term impacts.

### 4.2. Impacts on the Specification and Morphogenesis of Cardiac Lineages

Cardiac lineage specification is one of the initial and crucial phases of embryogenesis. It commences directly following gastrulation and is predominantly reliant on the integrity of spatiotemporal signaling cues. The cardiogenic mesoderm originates from the anterior lateral plate mesoderm and generates two significant progenitor fields: the FHF and the SHF [148]. These domains collaborate to construct the primitive heart tube and subsequently the four-chambered heart. Master TFs such as NKX2.5, GATA4, TBX5, ISL1, MEF2C, and HAND1/2 require not only precise activation but also proper orchestration via enhancers, chromatin accessibility, and epigenetic scaffolding.

Embryos produced with ART undergo in vitro procedures that subject the zygote and early cleavage-stage embryo to varying environmental circumstances, including elevated oxygen tension, mechanical rupture of the zona pellucida, extended culture durations, and serum-derived growth hormones. These alterations may induce epigenetic instability during pre-implantation development [149]. These pressures coincide with the epigenetic reprogramming wave, which eliminates DNA methylation marks from parental genomes and generates new ones de novo. The resetting phase is crucial for establishing gene regulatory networks specific to certain tissues, such as those determining the fate of mesodermal and cardiogenic cells. In this phase, alterations may induce prolonged transcriptional disarray, erroneous methylation at cardiac enhancers, and pathways that are only partially repressed or active [150].

Animal models corroborate these concerns. For instance, mouse embryos produced using IVF or ICSI and cultured in 20% oxygen have alterations in the expression of Hand1, Mef2c, and Nkx2.5 at the 8- to 16-somite stage, while appearing normal. These embryos have reduced H3K4me3 and elevated H3K27me3 at critical cardiac promoters, indicating a trend toward transcriptional repression [151]. This resembles issues associated with looping morphogenesis, hypoplastic ventricles, and abnormal patterning of the ventricular outflow system, particularly in SHF derivatives. In cardiac organoids produced from human embryonic stem cells, exposure to elevated glucose levels or oxidative stress disrupts WNT and BMP signaling, both of which are crucial for anterior mesoderm patterning and the proliferation of cardiac progenitors [152].

Furthermore, it is increasingly evident that non-coding RNAs, particularly miRs and lncRNAs, are pivotal in the regulation of cardiac morphogenesis. MicroRNAs such as miR-1, miR-133, miR-208, and miR-499 regulate myocyte proliferation, sarcomere formation, and the maturation of electrophysiological cells [153]. Research has shown that ART treatments can alter the miRNA profiles of preimplantation embryos and early fetal tissues. This may influence the post-transcriptional regulation of targets such as HDAC4, SRF, and TGFβR2, all of which are crucial for cardiac development. These little alterations may not result in apparent congenital anomalies, although they can increase the likelihood of the heart muscle experiencing hypertrophy, fibrosis, or conduction abnormalities over time [154].

Epigenetic misprogramming may disrupt left–right asymmetry, an initial phase of cardiac looping reliant on the expression of Nodal, Lefty, and Pitx2c. Data from ART animal models indicate reduced gene activity and an increased incidence of situs ambiguus or abnormal cardiac looping orientation. This indicates that the left–right axis may be particularly susceptible to early embryonic alteration in ART contexts. Maternal factors often overlooked, including COH, hormonal fluctuations, and cytokine modifications in the endometrium, may modify embryonic signaling environments and influence the specification of cardiac progenitors prior to implantation. Elevated maternal oestradiol levels during embryo transfer are associated with reduced BMP2/4 levels in the trophectoderm and mesodermal layers. This represents an additional correlation between ART-related treatments and cardiovascular issues.

In summary, ART influences cardiac development by altering transcriptional and epigenetic regulation in a complex and multifaceted manner. This includes alterations in chromatin remodeling at significant cardiac loci, anomalous enhancer–promoter looping, disrupted non-coding RNA networks, and modified upstream signaling gradients. These alterations not only elucidate the reasons behind the elevated risk of congenital heart disease and subtle myocardial dysfunction in ART offspring, but they also provide insights into potential preventive measures through improved culture systems, antioxidant supplements, or epigenetic biomarkers that can monitor the integrity of cardiac lineage in early embryos.

### 4.3. The Translational Significance of Epigenetic Biomarkers in Early Diagnosis

Epigenetic regulation is crucial for the accurate coordination of gene expression throughout embryogenesis, ensuring the temporal and spatial control of transcriptional programs vital for organogenesis. During cardiogenesis, DNA methylation, histone tail modifications, and non-coding RNAs work together to define cardiac lineage identification, chamber specification, and morphogenetic accuracy [155]. Epigenetic alterations may occur during the initial cleavage and blastulation phases of ART treatments, especially when environmental factors such as oxygen concentration, media composition, and mechanical stress diverge from physiological standards. Stable epimutations may result from these disturbances, disrupting early developmental processes and persisting into fetal and postnatal life, hence predisposing individuals to morphological and functional heart abnormalities [156].

An expanding corpus of information indicates that some epigenetic markers could serve as translational instruments for the early identification of embryos predisposed to cardiovascular abnormalities. Placental tissues and umbilical cord blood from children generated via ART exhibit anomalous methylation patterns in genes critical for heart development, such as GATA4, ISL1, NKX2.5, and HAND2. Included are promoter hypermethylation, hypomethylation of enhancer regions, and loss of imprinting, all of which may interfere with transcription factor binding, chromatin accessibility, and long-range enhancer–promoter interactions [133]. Distal regulatory elements of TBX5, a gene critical for septation and cardiac looping, have been linked to abnormal left–right patterning in animal models, indicating that methylation loss may act as a biomarker for conotruncal malformations.

Moreover, new advancements in the analysis of cell-free fetal DNA (cffDNA) during early pregnancy have improved the potential for non-invasive diagnosis of epigenetic abnormalities. To discover differential methylation in cffDNA at loci such as IGF2/H19, MEF2C, and ACTC1, which are implicated in epigenetic reprogramming and cardiac muscle structure, targeted bisulfite sequencing and methylation-sensitive digital PCR techniques have been utilized. The results suggest that cffDNA can be employed for epigenetic monitoring of heart development alongside aneuploidy screening, significantly enhancing the diagnostic capabilities of prenatal testing [157,158].

Simultaneously, circulating miRNAs are emerging as significant biomarkers due to their dynamic control throughout development, tissue specificity, and stability in biofluids. In the first trimester, maternal serum contains miRNAs such as miR-1, miR-133a, and miR-208a, which are crucial for cardiomyocyte differentiation, sarcomeric structure assembly, and electrophysiological system development. Irregular fetal cardiac shape has been associated with altered levels of these cardiac-specific miRNAs, potentially signaling early transcriptional dysregulation in the developing heart. Changes in miRNA profiles in maternal blood may occur before morphological problems are detected using fetal echocardiography, making them appropriate for early, non-invasive monitoring [159].

Moreover, investigations into blastocyst culture medium have indicated that the secretome may partially represent the epigenetic markers of the embryo. The gene expression pattern in the inner cell mass and trophectoderm is reflected by exosomal miRNAs and extracellular DNA fragments obtained from depleted media. Differential expression of miRNAs, such as let-7, miR-378, and miR-302, may offer indirect insights into cardiogenic potential or dysregulation, as these miRNAs are associated with early lineage segregation and organogenesis [160]. The prospect of using wasted media to non-invasively assess epigenetic health before embryo transfer is exceptionally exciting, especially within the framework of personalized ART.

These indicators may establish the basis for composite epigenetic risk scores for coronary heart disease in assisted reproductive technology pregnancies from a translational viewpoint. These scores may be utilized to categorize pregnancies into low, moderate, and high-risk classifications by integrating promoter methylation levels, enhancer activity, and circulating miRNA profiles. A multi-dimensional risk algorithm might be developed by integrating molecular data with clinical characteristics such as mother’s age, ovarian stimulation regimen, and embryo morphokinetics, thus enhancing embryo selection and pregnancy monitoring tactics.

Machine learning algorithms trained on comprehensive datasets of morphometric embryo data, epigenetic markers, and fetal outcomes may enhance predictive accuracy and aid therapeutic decision-making in the future. Artificial intelligence may excel at detecting nuanced epigenetic patterns or interaction networks that standard statistical methods overlook, facilitating earlier and more accurate interventions.

### 4.4. Prospective Approaches and Preventive Measures for Managing ART Embryos

As increasing data indicates that ART treatments alter epigenetic programming and elevate the risk of CHD, particularly in embryos subjected to suboptimal in vitro conditions, it is crucial to modify embryo handling practices informed by our understanding of molecular interactions. Preventive strategies should aim to minimize iatrogenic interference with developmental gene regulation, particularly during the preimplantation period when the epigenome is highly adaptable and dynamic. This phase, encompassing global DNA demethylation succeeded by de novo methylation, is crucial for establishing lineage identity and cardiogenic competence. During this period, any form of stressor, be it mechanical, oxidative, or chemical, can exert enduring effects on cardiac development, functionality in later life, and lineage allocation.

The primary concept of preventive intervention is to optimize culture media to the highest standard. Conventional media frequently contain unknown or animal-derived components that may lead to batch variability and oxidative degradation. Recent advancements indicate the utilization of chemically defined, xeno-free formulations that closely resemble the composition of human uterine and tubal fluids. These media are designed to maintain the stability of the body’s pH, osmolarity, and redox balance, thereby diminishing reactive oxygen species (ROS) that may influence the expression of critical cardiogenic regulators such as NKX2.5, MEF2C, and ISL1. The investigation also encompasses the incorporation of antioxidants (such as melatonin and glutathione precursors), metabolic regulators (including pyruvate and α-ketoglutarate), and epigenetic stabilizers (such as folates and vitamin B12) to assist with chromatin remodeling and the fidelity of DNA methylation.

Temperature stability, oxygen tension, and physical manipulation of the embryo are crucial for maintaining its developmental capacity. Elevated oxygen tension (about 20%), still utilized in numerous laboratories, is now recognized to induce oxidative stress, altering the histone acetylation profiles at cardiac enhancers and impeding the activation of HAND1 and GATA4 [156]. A transition to a low-oxygen culture (5%) resembles the intrauterine environment and has been associated with enhanced gene expression pertinent to cardiac development and a reduction in epimutations. It is crucial to minimize superfluous manipulation of embryos, such as ICSI pipetting force and laser-assisted hatching, as well as mechanical stress during biopsy procedures, particularly for blastocyst-stage embryos designated for transfer [161].

The real-time assessment of embryo health and epigenetic status at the molecular level is emerging as a revolutionary field of study. Non-invasive embryonic secretome profiling, which examines miRNAs, EVs, or DNA fragments in spent culture media, is a technology that may assist in identifying epigenetic dysregulation impacting early heart development [162]. Identifying downregulated miR-1 or aberrant methylation in TBX5 or ACTC1 within the secretome may indicate early dysfunction in myocardial lineage specification. Incorporating these molecular markers into embryo selection algorithms would enable prioritization of embryos exhibiting stable epigenetic profiles and a reduced risk of heart disease.

A secondary layer of protection comprises personalized ART techniques that consider the genetic and epigenetic profiles of both parents. Prior to conception, examining the methylation status at imprinted loci (such as IGF2/H19) and the integrity of mitochondrial DNA can indicate the potential vulnerabilities of parents to epigenetic instability. In women with polymorphisms in genes regulating methylation, such as DNMT1 and MTHFR, tailored dietary supplements and individualized stimulation protocols may mitigate ART-induced imprinting complications and subsequent cardiovascular difficulties. This approach aligns with the principles of developmental origins of health and disease (DOHaD), which emphasize the importance of maximizing health in early life.

In the future, epigenome-editing technologies such as CRISPR-dCas9 fused with DNA methyltransferase or demethylase domains may enable direct correction of aberrant methylation in critical cardiac loci within the preimplantation embryo. These approaches are not yet suitable for clinical application; nonetheless, they may rectify epigenetic damage induced by ART in vitro prior to implantation, restoring gene regulatory networks to their natural state. Simultaneously, organoid and embryo-on-a-chip systems are being developed to investigate the cardiotoxicity and epigenetic safety of culture additives and procedural modifications. These systems are excellent for enhancing procedures. To safeguard cardiac development during ART, we must shift our focus from solely assessing the morphology of embryos to also evaluating their molecular integrity. The most effective strategies to mitigate ART-related CHD involve preventive approaches centred on epigenetic stability, enhanced cultural systems, and individualized risk assessment. In the future, high-throughput omics, artificial intelligence, and real-time monitoring will likely converge to establish a new benchmark for the management of ART embryos.

Table 4 consolidates novel data regarding the molecular impacts of ART and underscores preventive strategies targeting essential regulators of cardiac development. For example, reducing oxidative stress through low-oxygen culture or the addition of antioxidants preserves the expression of NKX2.5, HAND1, and GATA4. Folate and B12 facilitate the DNA methylation processes essential for maintaining imprint stability at loci such as IGF2/H19 and TBX5. Profiling the embryo’s secretome, encompassing miRNAs such as miR-1 and miR-133, while preserving the embryo’s health, provides real-time insights into its cardiac condition. In the future, technologies such as CRISPR-mediated epigenetic editing and organoid-based toxicity assays may transform the assessment of preimplantation embryo quality by enabling the exact correction or prevention of epimutations that affect cardiac specification. These strategies indicate a novel ART paradigm grounded in molecular data that safeguards fetal development.

## 5. Conclusions

During the preimplantation period, ART methods such as ICSI, IVF, and in vitro extended culture under atmospheric O_2_ impose significant stress on cardiac precursors, potentially altering their epigenetic programming. These modifications influence the expression dynamics and regulatory integrity of essential cardiogenic transcription factors such as NKX2.5, TBX5, GATA4, ISL1, MEF2C, HAND1, HAND2, as well as contractile proteins, including ACTC1 and MYH6. This disrupts MESP1-mediated mesodermal lineage commitment and subsequent SHF–FHF patterning.

Disruption of DNMT1, TET3, and histone modifications such as H3K4me3 and H3K27me3 in cardiac enhancers alters chromatin accessibility at loci, including IGF2, H19, and HAND2, which are critical for looping morphogenesis and myocardial proliferation. Concurrently, oxidative stress generated by reactive oxygen species and components originating from xenobiotic media leads to the misregulation of miR-1, miR-133a, miR-208, and miR-499. These are recognized for their role in refining sarcomeric gene expression and facilitating Notch–BMP interactions. The ncRNA issues may be observed in both the trophectodermal vesicle cargo and the follicular fluid, indicating errors in the programming of early development.

Research involving animals and human cohorts indicates that ART is associated with a rise in the incidence of CHD, vascular stiffness, and hypertension during postnatal life. This indicates that the VEGF, eNOS, and Wnt/β-catenin pathways are compromised at an early stage. Alterations in imprinted clusters within the methylome, including KCNQ1OT1 and DLK1/GTL2, illustrate the impact of ART on the identification of cardiac progenitor cells and their metabolic priming throughout the organism.

To safeguard the integrity of cardiac development during ART, particular modifications are necessary: hypoxic (5% O_2_) culture, antioxidant co-treatment (such as MEL or GSH), defined xeno-free media to maintain DNMT activity, and vitamin optimization prior to conception (such as FA or B12). Furthermore, analyzing secretome-derived miRNAs and monitoring methylation in preimplantation embryos should facilitate the identification of epimutations in cardiogenic gene regulatory networks without necessitating dissection.

In the domain of ART, numerous practical consequences emerge from the mechanistic and translational results discussed below. Early postnatal echocardiographic assessment may facilitate the swift detection of mild circulatory alterations. Placental indicators, such as methylation patterns at imprinted loci, exhibit potential for identifying pregnancies at risk. Moreover, the optimization of embryo culture conditions—including oxygen concentration, media composition, and the incorporation of critical metabolites—remains a crucial area for improving both immediate and prolonged developmental outcomes. These metrics, grounded in mechanistic understanding, are effective methods to enhance ART practice and patient care.

In conclusion, cardiogenesis during ART is not merely a morphological result; it is also an epigenetically regulated process governed by the precise organization of transcription factor hierarchies, enhancer methylation, and miRNA dynamics. To ensure robust growth and optimal cardiovascular health in ART-conceived children throughout their lifetimes, forthcoming ART methodologies must incorporate molecular diagnostics and chromatin-based quality control.

## 6. Future Directions

Despite notable progress in elucidating the molecular and epigenetic consequences of ART, some key research gaps remain. A primary goal is the thorough identification of the most critical embryo culture factors—such as oxygen tension, media composition, and metabolite supplementation—that affect early developmental pathways and long-term health outcomes. Integrating embryo metabolomics into ART research may provide crucial insights into metabolic variables that predict implantation potential and subsequent health consequences. Single-cell epigenomics possesses the capability to deliver exceptional precision in delineating the regulatory landscapes of human preimplantation embryos. This will provide a more precise correlation between molecular modifications and developmental capacity. The advancement of non-invasive molecular embryo evaluation methods—employing discarded culture media, extracellular vesicles, or cfDNA—offers a strategy to improve embryo selection while maintaining embryo integrity. Addressing these gaps would enhance our comprehension of the impact of ART on individuals and facilitate evidence-based modifications to clinical practice, eventually benefiting patients and their offspring.

## Figures and Tables

**Table 1:** Key Genes Involved in Early Cardiogenesis and Their Vulnerability to ART-Associated Epigenetic Modifications.

Gene/Factor	Function in Cardiogenesis	Developmental Stage	Epigenetic Regulation	Potential ART-Related Disruption
**NKX2-5**	Master cardiac TF; initiates heart tube formation and chamber specification	Cardiac crescent to linear heart tube	Promoter methylation; interaction with GATA4 and T-box enhancers	Downregulation and enhancer destabilization in ART embryos
**GATA4**	Activates myocardial gene expression; interacts with NKX2-5 and TBX5	Early mesoderm to looping heart	Histone acetylation (H3K27ac) and coactivator recruitment	Reduced expression via altered HAT activity in ART blastocysts
**TBX5**	Regulates atrial/ventricular septation and limb-heart axis	Linear heart tube to chamber morphogenesis	Controlled by CREs with active histone marks	Reduced enhancer accessibility and misexpression in ART embryos
**ISL1**	Marks SHF progenitors; promotes OFT and RV development	SHF specification	Regulated by enhancer methylation and miR-17–92	Decreased expression and SHF mispatterning in IVF models
**MEF2C**	Required for RV and OFT formation; integrates Smad/TGFβ signaling	Looping stage	Direct target of FOXH1–NKX2-5 complex; Smad-dependent enhancer	Silencing of intronic enhancer via disrupted FOXH1 in ART embryos
**HAND1/2**	Ventricular morphogenesis and laterality; controls myocardial expansion	Chamber formation	Regulated by bHLH heterodimerization and methylation	Dysregulation linked to impaired chamber formation in IVC
**MYH6**	α-MHC; involved in early myocardial contraction and conduction	Chamber maturation	Isoform switch regulated by histone deacetylation	Altered isoform balance (MYH6/MYH7) in ART offspring hearts
**ACTC1**	Encodes α-cardiac actin; essential for sarcomere integrity	Early CM differentiation	Regulated by GATA/NKX co-binding sites	Impaired expression and sarcomere defects in ART-derived CMs
**DNMT3A/B**	De novo DNA methylation during implantation and germ layer formation	Zygote to blastocyst	Methylation of cardiogenic loci (e.g., NKX2-5, IGF2)	Aberrant methylation patterns in ART embryos
**TET1/2/3**	DNA demethylation and 5hmC production at developmental genes	Pre-implantation and epiblast stages	Regulates enhancer activity of cardiogenic TFs	Loss of 5hmC at heart enhancers in IVF embryos
**EZH2**	Mediates H3K27me3 silencing; maintains lineage boundaries	Gastrulation to organogenesis	Silences alternative lineage genes	Overexpression may repress cardiac mesoderm genes post-ART
**miR-1/133/208**	Regulate CM differentiation, proliferation, and hypertrophy	Cardiomyocyte lineage	Post-transcriptional repression of TFs (e.g., GATA4, SRF)	Dysregulated in ART placentas and fetal hearts

Important molecular controls of early heart development and how they might be messed up in embryos made using ART. The table shows important TFs, signaling components, and epigenetic regulators that control the development of the heart, its chambers, and its conduction system. A summary of each factor’s developmental time, mechanism of epigenetic control, and proof of ART-related dysregulation is given. These changes, which have mostly been seen in animal models and some human data, point to possible ways that ART techniques and CHD could be connected.

**Table 2:** Non-Coding RNAs Influencing Cardiac Architecture and Their Alterations in ART Embryos.

ncRNA	Type	Cardiogenic Role	Molecular Targets/Interactions	ART-Associated Disruptions
miR-1	miRNA	Promotes CM differentiation; represses inhibitors of sarcomeric gene expression	*Hdac4*, *Hand2*, *Klf4*	Downregulated in ART embryos; hypermethylated promoter
miR-133	miRNA	Maintains CPC proliferation; regulates cytoskeletal dynamics	*Srf*, *Ctgf*, *Cyclin D2*	Altered ratio with miR-1; affects CPC fate decisions
miR-17~92	miRNA cluster	Supports SHF CPC expansion and survival	*Bim*, *Pten*, *Tgfbr2*	Suppressed in IVF; associated with OFT malformations
miR-208a/b, miR-499	miRNAs	Regulate myosin isoform switching and ventricular identity	*Sox6*, *Sp3*, *Thrap1*	Histone modification imbalance; defective ventricular morphogenesis
Bvht	lncRNA	Primes mesodermal cells for cardiac lineage commitment	Interacts with *Swi/Snf* and *PRC2*	H3K27me3 enrichment suppresses expression in IVF embryos
Fendrr	lncRNA	Controls L/R asymmetry and posterior heart field patterning	*Foxf1*, *Pitx2*, epigenetic complexes	Repression in ART models linked to looping defects
Kcnq1ot1	lncRNA	Regulates imprinted cardiac loci and conduction pathways	Recruits *G9a*, *PRC2*; regulates *Cdkn1c*	Loss of imprinting in ART embryos; linked to abnormal cardiac growth
Circ-Sirt1	circRNA	Enhances CM survival under oxidative stress	Sponges *miR-93* → upregulates *Mef2c*	Disrupted *Qki*/*Fus* expression affects circularization in ART embryos
Neat1, Malat1	lncRNAs	Stress-responsive; linked to cardiac remodeling and fibrosis	Nuclear speckle organization; modulate splicing factors	Upregulated in ART-conceived cardiomyocytes; long-term cardiac remodeling

Legend: CM denotes cardiomyocyte; CPC signifies cardiac progenitor cell; SHF represents second heart field; OFT refers to outflow tract, and L/R indicates left–right.

**Table 3:** The Impact of ART on Epigenetic Regulators within Cardiac Gene Networks.

Epigenetic Regulator	Function in Cardiac Development	ART-Induced Disruption
DNMT1	Maintenance of DNA methylation during cell division; preserves methylation of cardiogenic gene promoters	Aberrant maintenance methylation leads to dysregulated cardiogenic gene silencing
DNMT3A/3B	De novo DNA methylation; crucial for cardiac lineage specification	Altered methylation patterns impair cardiac lineage commitment
EZH2 (PRC2 complex)	Histone methyltransferase that deposits H3K27me3, repressing non-cardiac genes in progenitors	Upregulation causes excessive repression of critical cardiac genes
KDM6A/KDM6B	Histone demethylases that remove H3K27me3, enabling gene activation in second heart field (SHF)	Downregulation results in persistent repression of cardiac transcriptional enhancers
BRG1 (SWI/SNF complex)	ATP-dependent chromatin remodeler that facilitates transcription of cardiac transcription factors	Reduced expression/activity impairs access to cardiac regulatory elements
CHD4 (NuRD complex)	Remodels nucleosomes to regulate chromatin compaction and cardiac enhancer accessibility	Mislocalization affects enhancer activity and gene silencing balance
H3K4me3	Activation mark at promoters of cardiac genes like NKX2-5, TBX5, MEF2C	Decreased enrichment at cardiac promoters delays gene activation
H3K27ac	Activation mark at enhancers; promotes transcription of heart-specific genes	Loss leads to insufficient enhancer activity and delayed transcriptional onset
miR-1	Suppresses HAND2; balances ventricular growth and cardiomyocyte differentiation	Downregulated in ART embryos; may cause myocardial hyperplasia
miR-133	Inhibits proliferation and promotes differentiation in cardiac progenitors	Suppressed expression results in altered cardiomyocyte proliferation and apoptosis
miR-208	Regulates cardiac myosin isoform expression and electrical conductivity	Reduced expression disrupts sarcomeric protein regulation and conduction

Table 3 enumerates the principal epigenetic regulators that govern cardiac gene expression during embryonic development. These include DNA methyltransferases, histone-modifying enzymes, chromatin remodelers, and non-coding RNAs, particularly microRNAs, which collectively regulate the transcriptional activation or repression of cardiogenic genes. The table delineates their physiological functions in cardiac development and the molecular alterations induced by ART-related therapies, including modifications in methylation dynamics, dysregulation of histone modifications, and aberrant microRNA synthesis.

**Table 4:** Preventive Measures in ART Embryo Handling and Their Molecular Targets.

Preventive Strategy	Targeted Molecular Pathways/Genes	Stage of Application
Low-oxygen (5%) culture conditions	Reduces ROS impact on NKX2.5, HAND1, GATA4	Preimplantation embryo culture
Antioxidant supplementation (e.g., melatonin, glutathione)	Prevents oxidative DNA/histone damage; stabilizes MEF2C, ISL1	Throughout embryo culture
Xeno-free, defined culture media	Reduces variability in cardiac enhancer activation	Preimplantation embryo culture
Folate and B12-enriched media	Supports DNMT1 activity; preserves methylation at IGF2/H19, TBX5	Preimplantation embryo culture
Minimized mechanical manipulation	Prevents disruption of cardiac chromatin dynamics	During ICSI, biopsy, hatching
Embryo secretome profiling	Monitors ACTC1, MYH6, and cardiogenic miRNAs in spent media	Day 3–5 blastocyst stage
Non-invasive miRNA screening	Detects dysregulation of miR-1, miR-133, miR-208 linked to myocardium	Day 3–5 blastocyst stage
Maternal methylation profiling	Identifies polymorphisms or methylation instability in parental genome	Preconception or before IVF cycle
CRISPR-dCas9 epigenome editing (future)	Corrects methylation errors in cardiogenic enhancers (e.g., HAND2)	In vitro (research setting)
Organoid/chip-based embryo testing	Assesses safety/toxicity of media components on cardiac gene expression	Protocol testing before clinical use

This table enumerates the principal methods to prevent ART from adversely affecting cardiac development. Each approach aligns with its biological goals, including transcription factors, signaling networks, and epigenetic regulators that govern the development and lineage commitment of cardiac cells. The table delineates the appropriate timing for each intervention, ranging from parental profiling before conception to monitoring at the blastocyst stage.
